# Millets: Journey from an Ancient Crop to Sustainable and Healthy Food

**DOI:** 10.3390/foods14101733

**Published:** 2025-05-13

**Authors:** Mrudula M. Mohanan, Akshitha Vijayakumar, Claus Heiner Bang-Berthelsen, Kiran Kumar Mudnakudu-Nagaraju, Radhakrishna Shetty

**Affiliations:** 1 Department of Biotechnology & Bioinformatics, JSS Academy of Higher Education & Research, Mysore 570015, Karnataka, India; mrudula51219@gmail.com (M.M.M.); akshitha.v@jssuni.edu.in (A.V.); 2 Research Group for Microbial Biotechnology and Biorefining, Research Group for Chemical Risk Assessment and GMO, National Food Institute, Technical University of Denmark, Henrik Dams Allé, 2800 Kgs. Lyngby, Denmark; claban@food.dtu.dk

**Keywords:** millets, sustainable food, gluten-free, health, nutrient-rich, climate-resilient

## Abstract

Millets, often known as “nutri-cereals”, have garnered renewed global interest due to their numerous health benefits, rich nutritional composition, resilience to extreme climatic conditions, and minimal environmental footprint. The advent of rice and wheat as staple foods in the 1960s led to drastic decline in millet cultivation worldwide. Recognizing the importance of millet, the United Nations (UN) declared 2023 as the International Year of Millets in an effort to accomplish Sustainable Development Goal 2 (SDG-2), i.e., zero hunger, by increasing millet production and fostering research and development to improve the integration of these grains into mainstream food systems. In recent years, global production of millets has surged, with India leading as the top producer. Millets are nutritionally advantageous, consisting of carbohydrates, antioxidants, and biologically active compounds such as flavonoids, carotenoids, phenolic acids, minerals, and vitamins. Incorporating millets into a balanced diet can help control and prevent diseases such as cardiovascular disease, diabetes, inflammation, and malnutrition due to their enriched vital nutrients, low glycemic index, and gluten-free nature. This indicates a transition of millets from an “orphan crop” to being used as ingredients for products (with or without fermentation) that are nutrient-rich, climate-resilient, sustainable, and health-promoting.

## 1. Introduction

As the world population is expected to reach 9.7 billion by 2050, the global food system faces critical challenges regarding nutritional security and environmental sustainability. This rapid expansion will be accompanied by changes in climatic conditions, resulting in food security challenges and the depletion of non-renewable resources [1]. In this contemporary setting, there is a demand for more food resources with high production, resilience to extreme weather, drought, and flooding, and the ability to improve soil quality and preserve ecosystems [2]. Given that millets are gaining popularity as a sustainable food source, they could help address future food security issues. The UN declared 2023 as “the International Year of Millets” due to their potential to solve concerns of nutrition, food security, and climate change in a sustainable manner [3,4]. With the goal of incorporating millet into major food systems in the future, these initiatives might greatly impact millet production and millet-based research and development.

Fostering millet farming could help achieve the SDGs as outlined by the UN. The “Zero Hunger Goal”, or SDG 2, aims to “end hunger, achieve food security, improve nutrition, and promote sustainable agriculture”. Similarly, the “Climate Action Goal”, or SDG 13, calls for “urgent action to combat climate change and its impacts” [5,6]. Millets are cultivated primarily as rain-fed crops with minimal or no use of fertilizer, and after harvesting, they can be stored for years without being harmed by pests. Compared to other major cereal grains, millets are less susceptible to insect attacks, indicating that no pesticide is needed for their cultivation, and they have lower global warming potential due to their low carbon footprint [4,7]. Moreover, they are traditional staple foods from ancient times with higher nutritional benefits [8]. Millets support environmental-friendly, sustainable agriculture since they can prevent the serious ecological harm caused by the indiscriminate use of pesticides and fertilizers [8,9,10]. Based on these considerations, millets have the potential to contribute to SDG-2 and -13. Likewise, millet cultivation and the expansion of millet-related value-added processing have the potential to contribute to SDG-1, the “No Poverty Goal,” by creating income in rural regions [4].

Millet, one of the earliest cultivated domestic crops from the family *Poaceae* (*Graminae*), is beneficial with regard to nutrition because of its high protein, carbohydrate, dietary fiber, minerals (calcium, magnesium, phosphorus, and iron), and vitamin content. In addition, these round-shaped small cereal grains are gluten-free and have a low glycemic index [11,12]. They are normally resistant to diseases and pests, can thrive on less fertile, dry soil, and can be harvested in 70 to 80 days. Their resistance to pests and diseases is due to various factors such as the presence of phenolic compounds that inhibit pathogens or pests, the hardness of millet grains which control pest infestation, and the presence of storage proteins (e.g., prolamins) in grains which act as a physical barrier and are indigestible by fungal or insect proteases [13]. Furthermore, millets can comply with climate change and outperform other grains like wheat and rice in terms of challenging growth conditions and nutritional value [14,15,16]. As a major cereal crop in the dry and semi-arid tropical regions of Asia and Africa, they serve as a staple food for human consumption as well as fodder for animals [17].

Millets are indigenous to various parts of the world and have been consumed by humans for more than 7000 years. Various millet species originated in different parts of the world, including northern China, Africa, the Middle East, and Europe, and then spread across the globe [18]. In addition, millets played a significant role in the diet of the prehistoric Indian, neolithic Chinese, and Korean Mumun communities. Proso millet is thought to be the first cultivated cereal grain and one of the earliest foods consumed by humans [19].

## 2. Rise of the Millet Era: A Historic View

The oldest known millet remains in the world were discovered in an early neolithic site of northern China, dated to approximately 8350–6750 BC; they were identified as proso or common millet. Later, foxtail millet content was identified in millet remains that were dated to after 6750 BC. Before rice and wheat gained widespread attention, millets were staple foods in the semi-arid parts of East Asia (China, India, Russia, Japan, and Korea) and still remain significant foods in those regions [19]. Furthermore, millets served as a staple food throughout the entire Eurasian continent. Evidence of the cultivation and consumption of millets (foxtail and proso millets) dating back to the Middle Jeulman pottery period (around 3500 BC) and in the Mumun period (1500–100 BC) in the Korean Peninsula has been reported by several studies [20,21]. In Japan, millets and their ancestors, such as barnyard and green foxtail grass, were cultivated dating back to the middle Jomon period (around 2500–1500 BC) [19,22,23].

Furthermore, anthropological data indicate that around 8000 BC, hunter-gatherers were familiar with wild varieties of sorghum. Sorghum was domesticated in the eastern Sudanese savannah in around 4000 BC, and it has spread to over 100 nations, serving as a staple food in a wide geographical area across the world (e.g., West Africa, India, China etc.) [24,25]. Archaeobotanical evidence showed the exploitation of pearl millet in the fifth millennium BC and domestication in the third millennium BC in northern Mali [26]. The finger millet crop was first domesticated in approximately 5000 BC in the highlands of Ethiopia and Western Uganda. It was subsequently brought to the Western Ghats of India in around 3000 years BC [27,28].

Ancient Indian texts like “Yajurveda” mention millets, such as finger millet (shyaamaka), barnyard millet (aanava), and foxtail millet (priyangava), which suggests that millet consumption was widespread in India and existed prior to the Indian Bronze Age, i.e., approximately 4500 BC [18,29]. On the Indian subcontinent, little millet is thought to have been domesticated around the early Harappan period (2600 BC), and Kodo millet around 3000 years ago [19]. The domestication of browntop millet (BTM) was also reported in India during the Neolithic-Chalcolithic period (~2800 BC) [29,30,31]. Table 1 represents the geographical and period of origin of different millets.

## 3. Overview of Global Production: Current Status

**Figure 1:**
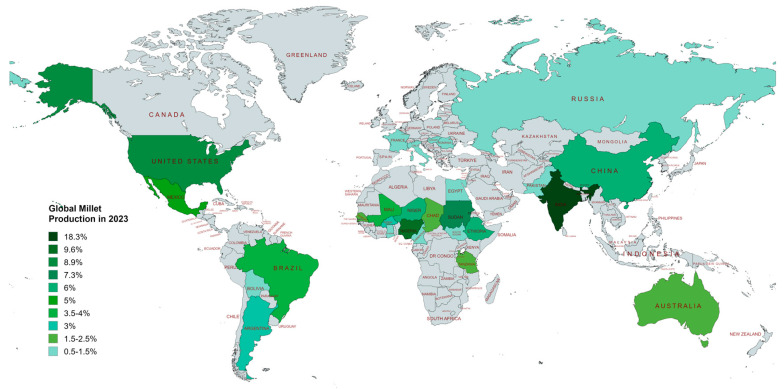
Top millet-producing countries across the world (Data source: International Production Assessment Division (IPAD), Foreign Agricultural Service, US Department of Agriculture https://ipad.fas.usda.gov/cropexplorer/cropview/commodityView.aspx?cropid=0459100, accessed on 5 July 2024; The data were calculated by combining the production of millets and sorghum across the world. The picture was created on mapchart.net.

Based on FAO data, millets are currently cultivated in more than 100 countries worldwide, with the top three producers being India, Nigeria, and the United States [42,43]. Among millets, the top-producing crop is sorghum, followed by pearl, foxtail, proso, and finger millets. According to a statistical analysis, the global millet cultivation area was estimated to have fallen by about 25.7% between the years 1961 and 2018, with the highest area decline in Asia and the lowest in Africa among the continents [17]. On the other hand, from 1961 to 2018, the global productivity of millet increased by 36%, from 575 kg/ha to 900 kg/ha. A comparative analysis of millet productivity among continents between 1961 and 2018 showed the largest increase in Europe (62%), Asia (56%), America (44%), and Africa (13%) [44,45]. Globally, 90.5 MMT (million metric tons) of millet were produced in the year 2023, of which India was the largest producer with 19% of total millet production, followed by Nigeria (10%) and the United States (US) (9%). The top millet-producing countries, along with their percentage of global millet production in 2023, are presented in Figure 1. According to the Observatory of Economic Complexity (OEC) data, global millet exports increased from $220 million to $258 million, representing a 1.13% growth, between 2022 and 2023. The leading millet exporters in 2023 were India ($41 million), followed by Uzbekistan ($40.5 million) and the United States ($33.8 million) [46,47,48]. Millet export and import data in 2023 are given in Table 2.

## 4. Millet Varieties: Diverse Options for Food

**Figure 2:**
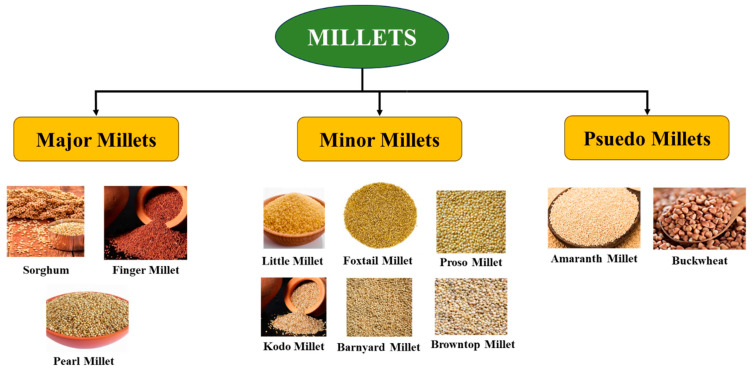
Different varieties of millets.

There are different types of millet, including finger, pearl, sorghum, little, foxtail, proso, kodo, barnyard, and browntop [49]. They are classified into major and minor millets based on their grain size. Additionally, there is another class known as pseudo-millets, which includes amaranth and buckwheat. Since the latter group does not belong to the *Poaceae* botanical family, which consists of ”real” grains, they are called pseudomillets [19,29]. However, they have comparable nutritional qualities such as high protein, dietary fiber, mineral, and vitamin contents, like true millets [50,51]. Figure 2 presents different varieties of millets.

### 4.1. Sorghum

Sorghum is one of the top five cereal crops in the world. Evidence from ceramic imprints from the Butana group site near Kassala suggests that sorghum domestication began in eastern Sudan in around the fourth millennium BC [52]. The Ethiopian highlands and semi-arid tropics across the world, i.e., in the Americas, Australia, the Southern High Plains of the United States, and Africa, and particular nations like China, Nigeria, Mali, and Cameroon, are the areas where sorghum is primarily cultivated [25]. It is a versatile crop, as it can be used not only for grain but also for sweet stem, fodder, and broomcorn. In addition, it can be utilized for the production of alcoholic beverages, building materials, bioethanol, and fuel [53]. Because of its ability to grow in difficult conditions with little access to water and nutrients, this crop is vital to food security in these regions [25]. According to Foreign Agricultural Service (FAS) data, 59.7 MMTs of sorghum were produced across the world in 2023, with the United States being the top sorghum producer at 14% of world production [48]. Among the major cereal grains, sorghum stands out as having significant concentrations of a wide range of bioactive substances that are uncommon in other cereals and that confer potential health benefits upon humans. Polyphenols, primarily flavonoids, and bioactive lipids, particularly phytosterols and policosanols, are the major bioactive components present in sorghum. In addition, sorghum endosperm typically digests starch more slowly than other cereals; this characteristic indicates the potential to regulate postprandial blood glucose levels in humans [54,55,56,57,58].

### 4.2. Finger Millet

It is generally accepted that finger millet, commonly referred to as ragi (in India) or dagusa (in Ethiopia), originated in the highlands of Ethiopia and Uganda. The continents of Asia and Africa are the largest producers of finger millet, and India is the world’s top producer [59]. Finger millet is the fourth most important millet in the world, behind sorghum, foxtail, and pearl millet. Red-colored finger millets, of which several types including yellow, red, tan, violet, white, and brown exist, are widely grown around the globe. With low to moderate rainfall and an altitude range of 500 to 2400 m, the crop is ideally suited to tropical regions and thrives in hot and dry conditions. Grain storage can keep the grains fresh for as long as fifty years [28,37]. Finger millet is distinct from other millets because of its five-layered testa (seed coat), which is a greater source of dietary fibers and micronutrients such as vitamins, minerals, and phenolic compounds. It contains 60% of the total millet polyphenols, which are present as glycosides [60]. Furthermore, it has been found that finger millet contains phenolic compounds, most of which are derivatives of benzoic acid, which possesses antioxidant properties [61,62,63].

### 4.3. Pearl Millet

Pearl millet, also known as bulrush millet, is primarily grown in Africa, Australia, and Asia. It is also known as “mahangu” in Namibia, “bajra” in India, “gero” in Nigeria, “hegni” in Niger, “sanyo” in Mali, and “dukhon” in Sudan. It is well-adapted to harsh weather conditions, such as rainfall of less than 250 mm and temperatures of approximately 30 °C [64,65]. According to [66], pearl millet exhibits a higher ceiling temperature (up to 42 °C) for grain yield in comparison to other cereals, rendering it a climate-resilient crop that is appropriate for cultivation in semi-arid regions across the globe. In addition, it possesses a number of benefits, including early maturity, resistance to drought, low input requirements, and resistance to most biotic and abiotic stresses [66,67,68]. Compared to other millets, pearl millet contains the maximum level of macronutrients and is significantly richer in soluble and insoluble dietary fiber and resistant starch, along with minerals such as potassium, phosphorus, manganese, copper, zinc, magnesium, and iron. In addition, it is a good source of calcium, folic acid, vitamin B (thiamine, riboflavin and niacin), and vitamin A [69,70,71].

### 4.4. Foxtail Millet

Foxtail millet, also called Italian millet, probably originated in China and is thought to have been domesticated during the neolithic age. It is one of the ancient crops cultivated in Europe and Asia. Foxtail millet (*Setaria italica*) got its name from the appearance of its seed head, which resembles a fox’s tail [23]. Since it is distributed throughout the world, it is known by several names, including Jopsal (Korea), Kazakh (China), German/Italian millet (Europe), Kakum or Kangni (India), and Tojara/Jawa/Batak/Jawae (Indonesia) [72]. It has a small genome size (approx. 515 Mb), a short life cycle (80–90 days), and is primarily abiotic stress-tolerant, particularly to drought and salinity, making it an excellent model for biofuel crops and genetic improvement programs [73]. The foxtail grain or seeds appear in a variety of colors, including black, brown, red, and orange or pale yellow. Since the grain is extremely small in size, a thousand grains constitute roughly 2 g of weight [74,75]. Since foxtail millet grains are coarse, only about 79% of the grain is digestible. The remaining non-digestible portion of the grain consists of some anti-nutritional ingredients and a comparatively high fiber content [76,77].

### 4.5. Proso Millet

Proso millet is cultivated extensively in China, India, Nepal, Russia, Ukraine, Belarus, the Middle East, Turkey, Romania, and the US [78,79]. Proso millet has several common names, depending on the geographical area where it is produced, such as common millet, hog millet, panivaragu, kashfi millet, broomcorn millet, French white, and red millet. It can grow in a variety of soil types and at altitudes of up to 3500 m in temperate climates. Growing most commonly as a late-seeded summer crop, proso millet serves as a summer annual grass that takes 60–100 days to fully develop [80]. The round grains measure approximately 3 mm in length and 2 mm in width, and they are covered with a smooth hull that can be grey, brown, black, yellow, white, creamy white, or red in color [33]. Multiple gene banks worldwide preserve about 28,000 proso millet germplasm lines, with the largest collection located in Russia [33,81,82]. They are rich in protein, minerals, vitamins, and phytochemicals, including phytate, flavonoids, and phenolic acids. In terms of anti-nutrients, proso millet does not appear to have the same protease inhibitory action as many other millets, although it does exhibit chymotrypsin inhibitory activity [83,84].

### 4.6. Kodo Millet

Kodo millet is produced mostly in Africa, China, India, Russia, and Japan and is classified as a coarse grain [85]. India is the leading producer of kodo millet, with 90% of global production. Although it takes a very long time to mature and has a very low yield (250–1000 kg/ha), it is well adapted to tropical and subtropical climates and has higher tolerance to biotic and abiotic stresses [80]. Its lower requirements enable this crop to flourish on soils with limited organic matter and low inputs, making it a perfect dual-purpose crop for rainfed regions [86,87]. Kodo millet is an annual grass plant with a maximum height of about 90 cm. The grains are covered in a hard husk, which makes them difficult to remove. The kodo millet color ranges from pale red to dark grey. The plant is known by several names, such as creeping paspalum, rice grass, Indian paspalum, kodo grass, ditch millet, and orvaragu [88,89]. With a higher level of proteins, vitamins, minerals, fiber, and sulfur-containing phytonutrients, kodo millet serves as a natural biofortified crop. However, its popularity is limited due to its low yield, lodging during harvest, market demand, and difficulties in dehulling. These issues make it necessary to use the most recent breakthroughs in breeding to create high-yield cultivars [89,90].

### 4.7. Browntop Millet

Browntop millet (BTM) is a warm season annual crop, with 12.13 g average yield per plant. It is mostly grown in China, Western Asia, Africa, Australia, and Arab nations [91,92]. BTM grows particularly well in semi-arid regions and at 2000–2500 m above sea level. It is highly resistant to drought, and its growth and maturation takes about 90 days. Due to its greater adaptability, BTM can be grown in both tropical and subtropical climates [39]. Tannins, flavonoids, resin, and quinones are the phytochemicals present in BTM. As a highly nutritious grain, 100 g of BTM provides 338 kcal of energy and contains a wide range of micronutrients, including phosphorus, zinc, magnesium, calcium, iron, and potassium. However, it is an underrated crop due to a lack of knowledge, widespread cultivation, and research [93]. Apart from its nutritional benefits, it also serves as an excellent substitute cover plant for managing soil erosion [94].

### 4.8. Barnyard Millet

Barnyard millet (*Echinochloa* species) is cultivated worldwide in warm and arid regions. It is most common in Asia, where it is cultivated in countries such as China, Japan, Korea, and India. The two primary species of barnyard millet are *Echinochloa esculenta* (Japanese barnyard millet) and *Echinochloa frumentacea* (Indian barnyard millet or Billion Dollar Grass), which are mainly cultivated for human consumption and animal feed [95]. Due to its great adaptability, barnyard millet can grow at altitudes of up to 2000 m above mean sea level in the summer. It grows fast among all small millets and completes its life cycle in 45–60 days, depending on the accession and growth environment, although it may take a prolonged period under the conditions of the Northern hill ecosystem of India [40,96]. Barnyard millet is an excellent source of vital nutrients, including dietary fiber, carbohydrates, and protein. Moreover, it differs from most cereal grains in terms of its higher concentrations of vital micronutrients, particularly zinc and iron. Phytic acid, flavonoids such as isoflavones and flavones, and phenolic compounds are the phytochemicals found in barnyard millet [97].

### 4.9. Little Millet

Little millet is widely grown as a cereal crop in western Myanmar, Nepal, Malaysia, China, and India. With 98% of the production area, India is the biggest producer of little millet. It serves as a significant crop in tribal agriculture in India’s eastern ghats, where it is widely used [80]. Sawa, Kutki, Samalu, and Samai are the common names for little millet. The crop is capable of being grown in harsh environments, including drought and waterlogging conditions, as it is well-suited to both dry and humid climates. It can reach maturity early [98,99,100]. Little millet is well-known for having high protein, phosphorus, and magnesium contents. In addition, it is a rich source of healthy polyunsaturated fatty acids (PUFA), gamma-aminobutyric acid (GABA), tocopherol (1.3 mg/100 g), carotenoids (51–104 μg/100 g), and flavonoids [101,102].

## 5. Nutrient Profiling: The Treasure from Millets

The nutritional value of millets is comparable to or better than those of many widely grown and consumed food grains such as rice, maize, and wheat. Millets contain a wide range of nutrients like dietary fiber, gluten-free protein, various antioxidants, vitamins, biologically active compounds, and minerals such as calcium, iron, zinc, phosphorus, etc. As such, these nutritionally rich ancient staples are also known as “nutricereals” [16,49,103]. The aforementioned nutrients are essential for maintaining different body functions, including immune system function and energy metabolism. Several epidemiological studies have shown the benefits of millet consumption in lowering the risk of diabetes, heart disease, and different types of cancers [104,105,106]. Millets have also been shown to boost immunity to respiratory infections, support healthy digestion, encourage detoxification, increase energy levels, and strengthen neurological and muscular systems. Adding millet to the diet can help address the issues generated by nutrient gaps and enhance general health [107]. Thus, as a possible source of vital nutrients, millets play an essential role in contemporary diets, particularly in developing and poor countries [103,108].

Based on the millet species, variety, crop management, and agroclimatic conditions, the carbohydrate content in millet ranges from 50–88%. Among the total carbohydrates in millets, starch constitutes up to 60–75%, followed by non-starchy polysaccharides (15–20%) and free sugars (1–3%). For comparison, wheat, rice, and maize contain 68–75%, 75.9–82.7%, and 63.19–74.5%, respectively [109]. Millets also consist of dietary fiber, composed of arabinoxylans, lignin, β-glucan, cellulose, and hemicellulose [110,111]. Dietary fibers are complex carbohydrates that are resistant to digestion and absorption in the small intestine and exert many health benefits. Improving gut health, lowering the risk of heart disease, preventing constipation, and lowering food glycemic index (GI) are a few of the health benefits of dietary fiber [112]. Additionally, dietary fiber directly affects the microbiota in the gut and produces short-chain fatty acids (SCFAs) by fermenting foods, which lowers the pH of the gut and provides protection against pathogen invasion and colonization [113]. Because of their advantageous effects, such as improving fecal bulk, shortening the time of intestinal transit, lowering cholesterol and glycemic levels, capturing potentially harmful substances (such as carcinogenic and mutagenic agents), encouraging the growth of the intestinal flora, etc., the consumption of these dietary fibers is linked to lowering the incidence of a number of chronic diseases [114,115].

When compared to other major grains, millets are well known for having a comparatively higher protein content, ranging from 5% to 17%, while wheat, rice, and maize contain 12–13.9%, 6.8–9.5%, and 8–9.0% protein, respectively. Among millets, proso millet is at the top, with a protein content of 9.5–17%, followed by sorghum (6.2–15.6%) and foxtail millet (8–14%) (Table 2). Of the total millet protein, 80% is present in the endosperm, 16% in the germ, and 3% in the pericarp [109,116]. In addition, essential amino acids, especially the limiting amino acids methionine, cysteine and lysine, are present in millets [49]. Millets are an excellent plant-based protein source for vegetarians and people from least-developed countries, because of their relatively high protein content, balanced amino acid profile, and good digestion [117,118]. Furthermore, millets primarily consist of unsaturated fatty acids, such as oleic and linoleic acid, with varying quantities of saturated fatty acids, including palmitic, stearic, and eicosanoids. In addition, minor quantities of bioactive lipids, including sterols, tocols, and oryzanols, also exist in millets as unsaponifiable lipid fractions. The presence of high amounts of unsaturated fatty acids and low-fat content in millets makes them a healthy option in a balanced diet [117,119].

In addition, millets are rich in B vitamins, including folate, pantothenic acid, thiamin, riboflavin, niacin, and vitamin B6, as well as minerals such as calcium, magnesium, potassium, phosphorus, manganese, and iron (Table 2). However, their concentrations vary among millets based on their variety, strains, and geographical area of production [120]. Vitamins and minerals are essential components of our diet due to their crucial roles in various bodily functions, including muscle and nerve function, hormonal regulation, maintaining the body’s water balance, and serving as the building blocks for our bones [117,121]. Millet grains also contain a variety of phytochemicals, such as phytate, phytic corrosive, policosanols, and phenolics, which are linked to antioxidant, anti-cancer, antihypertensive, and anti-diabetic, and anti-cardiovascular activities [122]. Among finger, kodo, pearl, foxtail, and little millets, proso millet has the lowest total phenolic content and kodo millet the highest [107,123,124]. The nutrient profiles of different millets, along with those of wheat, maize, and rice, are listed in Table 3.

Organic compounds present in food that restrict the digestion, utilization, and availability of minerals, dietary proteins, and carbohydrates are known as antinutritional components. The presence of dietary fiber, trypsin inhibitory factors, oxalates, phenols, tannins, and phytates in millets can also act as anti-nutrients due to their ability to impede enzymes and chelate metals [136]. However, according to scientific reports, anti-nutrients can lower the risk of various diseases, including inflammation, coronary heart disease, and breast cancer, when consumed in appropriate amounts [148]. In order to control the antinutritional properties and make millets fit for human use, basic processing methods such as dehulling, soaking, germination, roasting, drying, polishing, and milling (size reduction) are necessary [108,149]. In addition, millet-based value-added processed food items are produced through the application of modern or secondary processing techniques, such as fermentation, parboiling, frying, malting, puffing, extrusion, baking, popping, and flaking [108,150,151]. Fermentation helps to enhances food aroma, flavor, and texture, and also reduces the cooking time [152]. Parboiling, the process of soaking, steaming, and drying the grain before milling, affects the antioxidant properties and phenolic content [153]. Frying helps to increase the palatability [154]. Malting is known to enhance beneficial biochemical changes through the production of amylases and proteases, starch hydrolysis, etc. [155]. Extrusion (high temperature, short time processing) increases the bioavailability of minerals and shelf life [156]. Puffing deactivates the bacteria and improves the taste and flavor. Finally, popping, a common practice to prepare ready-to-eat food products, causes grain expansion and increases flavor [157]. Overall, the processing of millets helps to increase the bioavailability and utilization of nutrients

## 6. Nutrient-Rich Millets: A Way to a Healthy Life

### 6.1. Cardiovascular Disease

**Figure 3:**
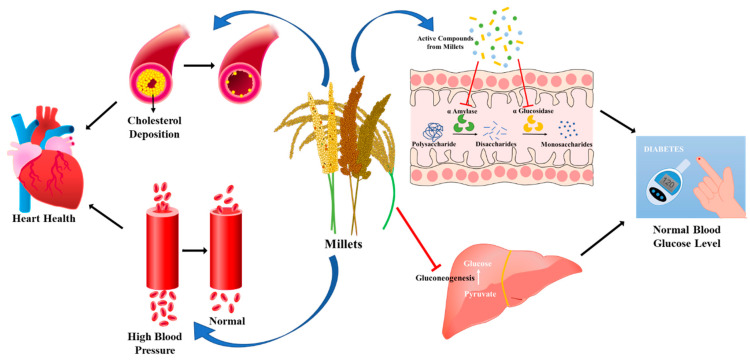
Effect of millets on heart health and blood glucose levels [162,165,166,167].

The majority of non-communicable disease (NCD) deaths, totaling 17.5 million, may be attributed to cardiovascular diseases [158]. A systemic review and meta-analysis performed from October 2017 to March 2021 by an international team of scientists found that millets can reduce the risk of developing cardiovascular disease by reducing total cholesterol, low-density lipoprotein cholesterol (LDL-C), and very-low-density lipoprotein cholesterol (VLDL-C), as well as triacylglycerol, in turn lowering systolic and diastolic blood pressure to normal levels and increasing high-density lipoprotein cholesterol (HDL-C) [159]. Rich in essential nutrients such as fiber, vitamins, and minerals, millets stand out as a valuable addition to a balanced diet. Their composition not only supports basic nutritional needs but also plays a crucial role in preventing and managing various health conditions. From promoting heart health and aiding digestion to managing diabetes and contributing to weight management, the diverse array of health benefits makes millets standout in the quest for optimal health [16,160]. The dietary fiber in millets contributes to a hypolipidemic effect, inhibiting atherosclerosis, and by binding to bile acids, it can reduce cholesterol reabsorption in the body. Consequently, the liver utilizes cholesterol to produce additional bile acids, resulting in a lowering of blood cholesterol levels [161]. A study conducted by Lee et al. in 2014 showed that the oil fractions separated from sorghum kernel significantly reduced non- high-density lipoprotein (HDL) plasma and liver cholesterol level in male hamsters fed with a high-fat diet [162,163]. In 2022, Yin et al. reported that supplementation of millet bran oil (MBO) in obese mice helped to alleviate hyperlipidemia, hepatic lipid accumulation, brown and white fat hypertrophy, plasma oxidative stress, hepatic lipid peroxidation, and hepatic oxidative stress. Moreover, MBO enhanced the number of certain benign gut bacteria, i.e., *Prevotellaceae* UCG_001 and *Akkermansia*, which play important roles in human metabolic regulation and health [164]. By reducing insulin resistance, improving glycemic management, lowering non-HDL cholesterol, and lowering blood pressure, and due to the presence of several antioxidants, millet can diminish the risk of atherosclerotic cardiovascular diseases (ASCVD) to some extent (Figure 3) [165].

### 6.2. Type 2 Diabetes Mellitus

Diabetes mellitus (DM) is marked by disruption in glucose homeostasis and imbalances in the carbohydrate, protein, and fat levels. Hyperglycemia not only signifies the presence of DM but also opens the door to the onset of various associated diseases [168]. The GI reflects food’s impact on blood glucose levels. Higher GI foods lead to rapid spikes in blood sugar, while lower GI foods induce gradual increases. With a GI score ranging from 40 to 70, millets exhibit lower values than wheat, refined flour, rice, and maize, rendering them a more suitable dietary choice for individuals with DM [169]. The mechanism by which millets guard against type 2 diabetes is linked to their low glycemic index and ability to diminish the postprandial blood glucose response [170]. By preventing hepatic gluconeogenesis, in one study, oral treatment with sorghum extract (0.4–0.6 g/kg body weight in rats) significantly decreased blood glucose levels in diabetic rats. They have suggested that the hypoglycemic effects shown by sorghum extracts might be linked to an insulin-independent mechanism, as studies on rat GLUT4 (glucose transporter type 4) translocation and Akt phosphorylation expression showed no significant glucose uptake by skeletal muscle [166]. Further, the soluble flavonoids and other phenolics in finger, Italian, and barnyard millet extracts showed strong inhibition of α-glucosidase and α-amylase activities when compared to the widely used anti-diabetic drug acarbose, suggesting that they might be able to lower postprandial hyperglycemia by delaying the breakdown of carbohydrates [169]. Treatment of type 2 diabetic mice with heat-treated foxtail millet starch and protein showed depletion of insulin and fasting blood glucose levels, an increase in the concentration of fecal short-chain fatty acids, and alteration in the composition of gut microbiota by enhancing the abundance of probiotics and decreasing harmful bacteria [171]. Whole grain proso millet (WPM) supplementation in Type 2 diabetic mellitus (T2DM) mice improved glucose tolerance, liver and kidney damage, and insulin resistance, as well as significantly lowering serum lipid levels and fasting blood glucose (FBG). The anti-diabetic effects of WPM function by enhancing the miRNA profile and activating the PI3K/AKT signaling pathway, which prevents gluconeogenesis (Figure 3) [172].

### 6.3. Immunomodulatory Effects of Millets

Recent research has suggested that millets have immunomodulatory effects, which can be beneficial in the prevention and treatment of various diseases [170]. A study conducted by Ji et al. in 2020 [173] demonstrated a reduction of TNF-α, IL-6, IL-1β, and nitric oxide (NO) by millet prolamin peptides (250 µg/mL) in lipopolysaccharide-stimulated macrophages. Since the millet peptides (200 µg/mL) were able to suppress p-IκB and p65, they could be an effective anti-inflammatory substitute. Millets are able to modulate immune signaling pathways, such as the nuclear factor-kappa B (NF-κB) and mitogen-activated protein kinase (MAPK) pathways. These pathways play a critical role in the regulation of the immune system. In another study by Cao et al., the immunostimulatory effects of finger millet extracted polysaccharide (6 μg/mL) were shown. Polysaccharide treatment on RAW 264.7 cells induced the production of proinflammatory cytokines such as IL-6, IL-10, TNF-α, and IL-1β, NO and activated the MAPK and NF-κB pathways [174]. The phenolic extracts of finger millets possess potential antiproliferative activity on breast cancer and hepatocellular carcinoma cell lines, as demonstrated by Zhang and Liu in 2015 [175]. By controlling the signaling pathways of NF-κB and MAPK, millet bran peptides can exert anti-inflammatory properties, which has been demonstrated both in vivo and in vitro, and a decline in inflammation in LPS-induced RAW264.7 cells [176]. The arabinoxylans (100 μg/mL) from little millet were found to function as an immunostimulant and showed the ability to alter the immune response in RAW264.7 cells by triggering the NF-κB and ERK signaling pathways. Meanwhile, the phenolic content (1.31 mg/g) of a kodo millet extract functioned as an immunomodulator by downregulating the NF-κB and ERK signaling pathways, which would modify the immunological response [177].

### 6.4. Prebiotic Properties of Millets

Prebiotics are indigestible food ingredients that probiotics utilize to produce health benefits for the host [178]. Prebiotics possess several crucial properties, including the ability to withstand digestion in the presence of gastric acidity and GI enzymes, resistance to absorption in the upper GI tract or large intestine, and selective utilization by the probiotic gut microbiota. This microbiota generates a variety of enzymes that hydrolyze prebiotic carbohydrates, ferment-oligosaccharides, and form SCFAs [179,180,181]. Due to their high content of dietary fiber and phenolic compounds, millets are known to impose modulatory effects on gut microbiota, which have been shown to improve gut health by increasing the population of beneficial bacteria such as *Lactobacillus* and *Bifidobacterium* [182,183,184]. Pure cultures of *Lactobacillus* and *Bifidobacterium* have been observed to break down 60–80% of the pearl millet fiber, releasing the SCFAs acetate, propionate, and butyrate. Further, the authors of [185] used kodo, foxtail, and barnyard millets to make a prebiotic multigrain beverage with higher antioxidant activity and low GI. In another study [186], a millet soup recipe from kodo, foxtail and barnyard millets achieved a high prebiotic activity score, strong antioxidant activity, and a low GI [184,185,186]. In a preclinical study on mice models conducted by Chen et al., consumption of foxtail millet porridge was shown to reduce constipation, increase gastrointestinal motility, and improve the gut microflora (with an increased proportion of *Bifidobacterium* and *Lactobacillus*) due to its potential prebiotic efficacy [187]. The potential role of germinated pearl millet in the improvement of gut health by increasing the thickness and depth of the crypt, goblet cell count, and propionate concentration was demonstrated by Theodoro et al. in high-fat, high-fructose (HFHF) diet-fed rats. In addition, germinated millet improved the gut microbiome by reducing Desulfobacterota phylum and *Oscillibacter* genus and increasing *Firmicutes*, *Bacteroidetes*, and *Actinobacteria* [188].

### 6.5. Antioxidant Properties of Millets

Millets exhibit excellent antioxidant activity due to their high content of phenolic compounds, flavonoids, tannins, carotenoids, tocopherols, and other bioactive molecules, which contribute to their potential health benefits, including reducing the risk of chronic diseases and supporting overall health [189]. They are also rich sources of vitamin E and minerals. Studies have suggested that vitamin E has a variety of health benefits, including improved immune response and cell-mediated immunity, antioxidant activity that provides protection against cell damage and degenerative diseases, and the potential to reduce the risk of chronic diseases and cardiovascular events [190,191]. An analysis of how different processing techniques affect the antioxidant activity of pearl millet revealed that the bran-rich fraction had increased antioxidant activity because of its high levels of phytic acid, tannin, and flavonoids [192]. In comparison to proso and foxtail millet, finger millet exhibited higher levels of phenolic content and antioxidant activity. The variety and cultivation location of millets has an impact on the phenolic content and antioxidant activities [193]. The phenolic content and antioxidant activity of millet grains are significantly impacted by dehulling. Phenolic extracts of processed millets generally show antioxidant activity in the following order: millet husk > whole grain > dehulled grain > cooked dehulled grain. Studies have shown that the level of antioxidant activity of whole grains and dehulled grains varies among different millet varieties and under different physiological conditions [194,195].

## 7. Conclusions

According to archaeological evidence, millets have been domesticated since around 8050 BC in the semi-arid regions of Asia and Africa and subsequently spread to different continents, including Europe. The advent of rice, wheat, and maize made millet an underrated crop; however, the quest for a more sustainable, healthy, and nutritious crop has brought it back into the mainstream. Total millet production data for 2023 indicate that India is the greatest producer, accounting for 19% of the global millet production, followed by Nigeria with 10% and the United States with 9%. Millets present a compelling solution to the dual problems of fighting lifestyle-related health diseases and achieving environmental sustainability. They promote biodiversity and sustainable agriculture because they are climate-resilient crops that require minimal water and chemicals. Millets have protective benefits against diabetes, cardiovascular disease, and obesity because they are high in fiber, vital micronutrients, and bioactive substances. They are also appropriate for people with celiac disease or gluten intolerance because they are gluten-free. Beyond improving health, millet farming supports rural livelihoods, resurrects traditional agricultural methods, and empowers smallholder farmers. However, increased knowledge, policies that support them, and advancements in processing and marketing are required for this crop to reach its full potential. Therefore, millets could become staple foods by being incorporated into regular diets through institutional initiatives, public awareness campaigns, and creative cooking. To conclude, the development of novel, value-added, millet-based food products through secondary processing such as fermentation, baking, malting etc., may help to establish a millet-based diet trend in the global population.

## Figures and Tables

**Table 1:** Different types of millet and their place and time of origin.

Millet		Origin	Period	References
Common Name	Scientific Name			
Foxtail millet	*Setaria italica*	Northern China	Neolithic period (9050 to 7050 BC)	[32]
Proso/Common/Broomcorn millet	*Panicum miliaceum*	Northern China	Neolithic period (8350–6750 BC)	[23,33,34]
Sorghum	*Sorghum bicolor*	Northeast-central Africa	Neolithic period (8000 BC)	[24,25]
Kodo millet	*Paspalum scrobiculatum*	India	Iron age (977 BC)	[30,35,36]
Finger millet	*Eleusine coracana*	Eastern Africa (Western Uganda to Ethiopia)	Iron age (2977 BC)	[28,30,37]
Pearl millet	*Pennisetum glaucum*	Northeast Mali	Middle Holocene (~5000 BC)	[26,38]
Little millet	*Panicum sumatrense*	India	Early Harappan period (3300–2600 BC)	[29,31]
Browntop millet	*Brachiaria ramosa*	India	Neolithic-Chalcolithic period (~2800 BC)	[39]
Japanese Barnyard Millet	*Echinochloa esculenta*	Japan	Yayoi period (5000–4000 BC)	[40]
Indian Barnyard Millet	*Echinochloa frumentacea*	India and Africa	5000 BC	[40,41]

**Table 2:** Export and import data of millets in 2023 [47]; * M—million.

Exporters		Importers	
Country	Trade Value (USD)	Country	Trade Value (USD)
India	41 M *	Pakistan	37.1 M
Uzbekistan	40.5 M	Indonesia	31 M
United States	33.8 M	Germany	12.4 M
Russia	29.2 M	Belgium	12 M
Ukraine	23.9 M	United Arab Emirates	8.74 M
France	14.4 M	Italy	8.25 M
China	11.1 M	Turkey	8.19 M
Tanzania	8.87 M	United Kingdom	8.02 M
Poland	8.7 M	Canada	8.01 M
Turkey	6.16 M	Kenya	7.81 M
Netherlands	4.41 M	Japan	6.4 M
Germany	4.39 M	Netherlands	6.03 M
Ethiopia	3.15 M	Nepal	5.44 M
Canada	3.1 M	Morocco	5.19 M
Austria	2.57 M	Malaysia	4.95 M
Argentina	2.14 M	Spain	4.67 M
Belgium	2.11 M	United States	4.35 M
Kazakhstan	1.96 M	Philippines	4.18 M
South Africa	1.28 M	Iraq	4.05 M
Uganda	1.14 M	Saudi Arabia	3.84 M
Bulgaria	1.04 M	Thailand	3.84
		Poland	3.77 M
		Israel	3.75 M
		Senegal	3.74 M
		South Korea	3.45 M
		Libya	3.06 M

**Table 3:** Comparison of nutrient composition of millets with maize, wheat and rice.

Millets	Carbohydrate (%)	Dietary Fibre (%)	Proteins (%)	Fat (%)	Vitamins (mg/100 g)	Minerals (mg/100 g)	References
Sorghum	67.6–79	10.2	6.2–15.6	1.5–4	Riboflavin—0.14, Thiamine—0.09, Niacin—2.8	Calcium—27–28 Iron—4.4 Phosphorus—222–287 Sodium—7 Potassium—249	[108,117,125]
Finger millet	65–77	4–20	5–12.7	1–2	Riboflavin—0.19–0.33 Thiamin—0.33–0.48	Calcium—240—344 Iron—3.5–6 Zinc—1–2 Phosphorus—150–300 Potassium—350–550 Manganese—5–6 mg	[107,117,126,127,128]
Pearl millet	61–62	8–19.5	9–13	1.5–7	Riboflavin—0.2 Thiamine—0.25 Niacin—0.9 Vitamin E—2	Calcium 27.4—48.6 Iron—6.4–16 Zinc—2.7 Phosphorus—289 Magnesium—124	[16,68,117,129]
Foxtail millet	60–75	14	8–14	3–5	Folate—42 Vitamin E—31	Calcium—30–50 Iron—3–5 Zinc—2–3, Phosphorous—200–300, Potassium 250–400, Sulfur—100–200, Magnesium—40–150	[72,107,117,130,131,132]
Proso millet	68.2–70	8.5—12.5	9.5–17	1.1–3.5	Riboflavin—0.29 Thiamine—0.42 Niacin—4.72	Calcium—8–14 Iron—2–3 Zinc—1.68–2 Phosphorus—206–285 Magnesium—114 Potassium—195	[124,133,134,135]
Kodo millet	63–66.6	6.39–15	8–9	1.4–2.55	Riboflavin—0.09–0.2 Thiamine—0.15–0.29 Niacin—1.49–2	Calcium—15–27 Iron—0.5–2.34 Zinc—1.65 Phosphorus—101–188 Magnesium—122–147 Potassium—188 Sodium—3.35 Manganese—0.33	[85,136,137]
Browntop millet	71.32–72	8.5–12.5	8.98–11.5	1.89–4.88		Calcium—28 Iron—7.72 Zinc—2.5 Phosphorus—276 Magnesium—94.5 Potassium—60 Sodium—7.60 Manganese—1.99 Copper—1.23	[93,138]
Barnyard millet	51.5–66	6.4–31.7	5–14	2.5–6.3	Riboflavin—0.1 Thiamine—0.4 Niacin—4.2	Calcium—14–22 Iron—15.6–18.6 Zinc—4.9 Phosphorus—121 Magnesium—86.2 Manganese—0.7 Copper—0.6	[40,95,107,108]
Little millet	65.55	7.72–12.2	7.7–10.13	3.89–4.7	Riboflavin—0.05–0.09 Thiamine—0.26 Niacin—1.29–3.2	Calcium—16–17 Iron—1.26–9.3 Zinc—1.82 Phosphorus—130–220 Magnesium—91.41–133 Potassium—220 Sodium—4.77 Manganese—0.23	[136,137]
Maize	63.19–74.5	6.69–13.17	8.31–9.29	3.29–4.25	Riboflavin—0.08 Thiamine—0.3 Niacin—1.9	Calcium—10 Iron—2.3 Zinc—0.49–8.66 Phosphorus—348 Magnesium-139 Potassium—286 Manganese—0.35–0.47	[109,139,140,141,142]
Wheat	68–75	1.2–2.9	12–13.9	1.3–3.1	Riboflavin—0.07 Thiamine—0.26 Niacin—2.0	Calcium—41 Iron—5.3 Zinc—4.6–5.8 Phosphorus—299.6–357.4 Magnesium—107.9–117.3 Potassium—324.8–358.7 Manganese—1–1.2	[109,143,144,145]
Rice	75.87–82.70	0.2–0.85	6.8–9.51	0.6–0.92	Riboflavin—0.03 Thiamine—0.12 Niacin—1.5	Calcium—23–28 Iron—0.8–1.4 Zinc—1.1–2 Phosphorus—115–223 Magnesium—25–143 Potassium—115–223 Manganese—1.1–3.7	[109,143,146,147]

## Data Availability

No new data were created or analyzed in this study. Data sharing is not applicable to this article.
